# Discrepancies in Data Reporting for Rabies, Africa

**DOI:** 10.3201/eid1904.120185

**Published:** 2013-04

**Authors:** Louis H. Nel

**Affiliations:** University of Pretoria, Pretoria, South Africa

**Keywords:** rabies, rabies virus, viruses, reportable diseases, zoonoses, incidence, data reporting, discrepancies in data reporting, Africa

## Abstract

Human rabies is an ancient disease but in modern times has primarily been associated with dog rabies–endemic countries of Asia and Africa. From an African perspective, the inevitable and tragic consequences of rabies require serious reflection of the factors that continue to drive its neglect. Established as a major disease only after multiple introductions during the colonial era, rabies continues to spread into new reservoirs and territories in Africa. However, analysis of reported data identified major discrepancies that are indicators of poor surveillance, reporting, and cooperation among national, international, and global authorities. Ultimately, the absence of reliable and sustained data compromises the priority given to the control of rabies. Appropriate actions and changes, in accordance to the One Health philosophy and including aspects such as synchronized, shared, and unified global rabies data reporting, will not only be necessary, but also should be feasible.

Rabies, despite its high case-fatality rate and preventability (through efficacious preexposure and postexposure prophylaxis), has in recent years progressively become established as a neglected disease, and most human cases are associated with dog rabies endemic to countries in Africa and Asia. However, there are numerous other serious infectious diseases that, like rabies, are underreported and linked with poverty in the developing world. How then should these diseases be prioritized?

This report presents considerations that influence the priority status of rabies, as well as issues that could differentiate rabies and should play a role in establishing the relative role of this disease in Africa and other parts of the developing world. It also discusses 1 key area that needs to be addressed before a bona fide demonstration of rabies incidence and progress toward effective dog rabies control will be feasible. This area is the need for true cooperation and synergy between global organizations and national authorities with respect to responsibilities related to effective and thorough surveillance with synchronized and responsible data reporting. Analyses of examples from Africa indicate that the above aspects are seriously compromised.

## Factors Leading to Complacency and Neglect of Rabies

Rabies virus, a classical zoonotic pathogen, has an extensive host range and can probably infect all terrestrial mammals. Although vampire bat rabies has a major effect with regard to livestock losses in Latin America (*1*), rabies is generally not associated with agricultural animals because the main terrestrial reservoirs are domestic dog populations of the developing world and wildlife carnivores elsewhere. Thus, rabies is often handled unconnectedly by health and veterinary authorities, and there is regular confusion as to who is responsible for controlling this disease. It is also likely that public demand for effective control measures would have been far greater if rabies had been a major disease of economically vital animals with a corresponding effect on livelihood. For Africa, a case in point is rinderpest, a viral disease for which there had been, figuratively speaking, as many eradication campaigns as pandemics. These campaigns were typically pan-African and driven by considerable international support and cooperation (*2*).

The history of rabies in Africa is not well recorded, but it is well accepted that the disease must have been present in in northern Africa for hundreds of years, particularly as an urban disease of dogs and also associated with cycles in the Middle East. Rabies became epizootic in many countries of sub-Saharan Africa only during the nineteenth and twentieth centuries; in this region, the disease became well-established in dogs and involved wildlife species over large areas (*3*).

Today, no regions of or countries in mainland Africa are known to be free of rabies (*4*). In addition, Africa harbors several rabies-related viruses. Historically, the isolation and epidemiologic analyses of these viruses largely correlated with specific surveillance studies or diagnostic competencies (*5*). This scenario is also true for the most recent discoveries of 2 novel African lyssaviruses (*6*,*7*), and all indications are that other as yet unknown lyssaviruses remain to be found. Without more comprehensive and routine surveillance and laboratory-based diagnosis, the epidemiology and the potential role of these viruses or emergence of these viruses remain speculative. Unfortunately, a lack of consistent and sustained or routine laboratory-based diagnosis for rabies (and no capacity to distinguish rabies-related viruses) may well be the status quo in many countries in Africa.

In humans, rabies often develops with a wide variety of nonspecific clinical symptoms, and symptoms believed to be typical of rabies (e.g., foaming at the mouth, hydrophobia, and extreme aggressiveness) are frequently not observed. Approximately 30% of human rabies cases develop in the paralytic or dumb form (*8*), and the overlap of symptoms with those of other infections often leads to misdiagnosis (*9*–*12*). Apart from misdiagnosis, rabies exposures are often ignored or deemed as minimal in dog rabies–endemic areas of the world. Because dogs are common as companion animals in most cultures, the exposure risk is actually greater than for many other zoonotic diseases. However, postexposure prophylaxis is unlikely to be sought after lick-associated exposures to dogs. Some cultures are known to believe that the lick from a dog is useful for wound treatment (*13*). In conjunction with this belief, some cultures believe that the aggressive and uncharacteristic behavior of persons or animals with symptoms of rabies is caused by sorcery or demon possession. Far too often rabies patients end up at tribal or traditional healers whose treatments include exorcism, administration of toxic herbs, and other such undesirable interventions (*14*,*15*).

Rabies is one of the oldest recognized diseases in human history, and there is anecdotal evidence of its presence in Mesopotamia and elsewhere in the Mediterranean basin since antiquity (*16*). Because this evidence has been known for many years, an unfortunate consequence has been a loss of newsworthiness, which has compromised awareness and priority in public and professional practice. A contrasting example could be witnessed with the newly emerged influenza A(H1N1)pdm09 virus, which caused widespread panic and has received much attention over the 3 years since its emergence. In the 17 months from April 2009 through August 2010, a total of 18,000 deaths caused by swine-origin influenza (mostly associated with other primary risk factors) were recorded worldwide (*17*). In contrast, rabies is conservatively estimated to cause ≈50,000 deaths per year, mostly in children, and is not associated with any other health risk factors (*18*).

## Global and Regional Structures and Reporting of Rabies Cases

The World Health Organization (WHO) has reported that rabies has the highest case-fatality rate of all infectious diseases of humans (*19*), and most human exposures occur in children <15 years of age. For this reason, WHO has deemed rabies a reportable disease. Although WHO recognizes rabies as a reportable disease, many countries (e.g., India) (*20*) do not.

To attempt an assessment of the incidence of this disease globally, WHO has collected rabies data since 1959 and in the late 1990s created and administered Rabnet, a rabies-dedicated Web platform, to which countries have been requested to submit annual rabies statistics (*21*). These figures were published under various categories, including human cases, animal cases, presence or absence of rabies, national rabies vaccine production and importation, and rabies vaccine administration. In addition, there were several subsections, which enabled specification of certain criteria. For instance, under animal rabies, one could choose total number of dog cases and further specify whether one would want to observe dog rabies positive, dog rabies negative, or both. Figures obtained from Rabnet were frequently used in publications and in country reports. Despite having been a worthwhile undertaking, the Rabnet website has been closed indefinitely (until further notice) since late 2011, given the realization of incorrect reporting and to avoid subsequent misrepresentation.

The World Organisation for Animal Health (OIE) also regards rabies as a reportable disease, and OIE statistics for such diseases are published on the World Animal Health Information Database (*22*). The structure of this database includes 1) immediate notifications and follow-up reports submitted by member countries in response to exceptional disease events occurring in these countries and follow-up reports about these events; 2) reports every 6 months describing OIE-listed disease situations in each country; and 3) annual reports providing further background information about animal health, and laboratory and vaccine-production facilities. Thus, information for specific diseases is available on the website, and one can compare multiple countries with each another. Reports are submitted biannually by each country, and a final report is issued at the end of each year. The information for rabies gives detailed monthly case reports of rabies in animals for every month of each year and, in some cases, for each district or province of the country.

The Southern and Eastern African Rabies Group (SEARG), founded in 1992, focuses on control of dog rabies in countries in Africa. Official meetings are held approximately every 2 years, at which representatives from member countries gather and present data regarding rabies in their country in standardized country reports. The information from these reports is published on an open-access website (www.searg.info). The country reports focus on human and animal rabies (domestic and wildlife) and request information regarding vaccine purchases and/or production and vaccination strategies.

## Discrepancies and Deficiencies in Rabies Reporting

**Figure 1:**
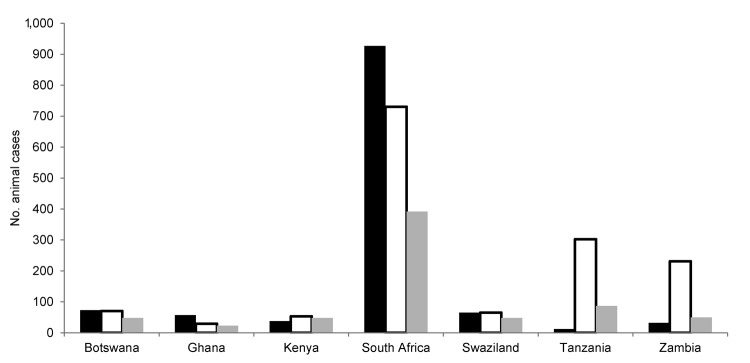
Number of rabies cases in animals reported in 2007 from countries in Africa classified as developing countries. Data were obtained from Southern and Eastern African Rabies Group reports (black bars), the World Health Organization (Rabnet) (*21*) (white bars), and the World Organisation for Animal Health World Animal Health Information Database (gray bars).

**Figure 2:**
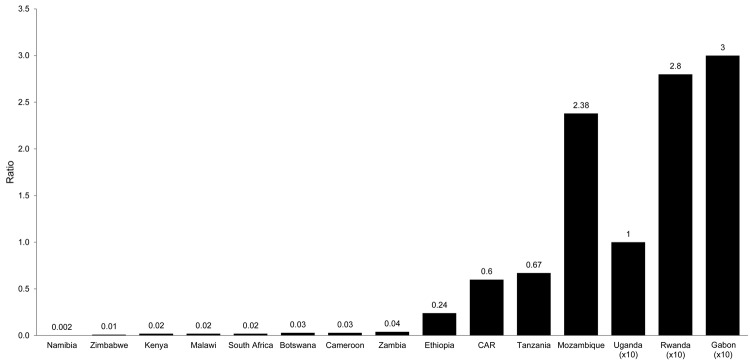
Ratio of human to animal cases of rabies reported in 2010 from Southern and Eastern African Rabies Group countries. Ratios are indicated above the bars. CAR, Central African Republic.

Lack of reporting on rabies data by most developing countries is disconcerting. Examination of data on WHO and OIE web sites showed that information was frequently missing for an entire year or more in several countries. Data from selected countries from the southern African region for which SEARG, WHO, and OIE data reports were available are shown in Figure 1. Data submitted to the 3 authorities varied considerably in all examples. The only data from any of the countries that showed some correlation was that of Swaziland, where data submitted to SEARG and WHO were the same, but data submitted to OIE were different. In addition, when the ratio of human to animal rabies cases was analyzed, a vast range of ratios was typically found for data for 2010 (Figure 2). Data for individual countries ranged from high ratios of human rabies cases to animal rabies cases to (the more rational) low ratios of human cases to animal cases. Some countries reported only human rabies cases (clinical diagnoses for most countries in Africa). Such inconsistent data can be considered a further indicator of poor surveillance practices.

Inconsistencies in reporting of rabies epidemiologic information to WHO and OIE did not apply only to developing countries. Although inconsistencies were not as great as those observed in developing countries, which was likely caused by the fact that rabies cases are less frequent because of adequate control measures, they were still evident from the industrialized world. Also, for various countries, data for specific years were submitted only to OIE, only to WHO, or to neither organization.

Discrepancies for rabies epidemiologic data between various authorities could be interpreted as symptomatic of a larger problem, which should be addressed on a global scale. One likely reason for the lack of consistency of rabies data is the different focus areas of WHO and OIE. WHO focuses mainly on human disease, and will most likely receive rabies data from medical health authorities. In contrast, the animal disease focus of OIE suggests that veterinary services will submit rabies data to this body. Inconsistencies described in this report suggest a need for improved collaborative effort and effective communication between all relevant authorities with regard to diseases that simultaneously effect human and animal health. The imminent neglect of any zoonosis of which the main reservoir host is not an economically vital animal species is predictable unless addressed by unconditional execution and instruction of the One Health paradigm on global, regional, and country levels.

## Conclusions

Rabies remains endemic throughout Africa, and for all the reasons discussed, loses visibility in Africa because it typically oscillates disconnectedly between authorities concerned with either human or animal health. Poor epidemiologic surveillance and inconsistent reporting, including that to responsible global authoritative bodies, has created a lack of rabies awareness and appreciation of its effect on humans in Africa. The absence of reliable and sustained rabies data compromises the priority that the control of rabies should be given, considering that a lack of laboratory-based proof of disease incidence will innately counteract attempts to justify (on national levels or to global funding agencies) the need for extensive and expensive rabies elimination programs.

OIE has recently released its Fifth Strategic Plan, which includes the One Health approach and is committed to improved cooperation with human–animal–environment interfaces (*23*). Despite this fact, the forms required by each of the organizations to be completed (e.g., for rabies) are different. This difference creates additional work for the authority responsible for submission of data and can lead to inconsistencies. If submission forms are standardized, then the same form can be sent to the various organizations. Another alternative would be to have 1 body to which to report epidemiologic data to, and from this body the major organizations can use and publish the appropriate data. This uniformity will prevent submission of inconsistent data but will require true collaboration between medical and veterinary sectors (the One Health approach). Because of the need for consistent and transparent data, appropriate actions and changes, in accordance to the One Health philosophy, will be necessary and feasible.

On a continental level, the One Health approach has already been shown to be beneficial toward rabies control in at least 1 part of the developing world, when implemented by the Pan American Health Organization in Latin America (*24*). In contrast, there is no pan-African approach to rabies control, although small regional efforts present hope. The rabies control program in Kwa-Zulu Natal in South Africa (*25*), which is supported by the Bill and Melinda Gates Foundation, celebrated a year free of human rabies on June 24, 2011. That occasion constituted the first time in 20 years that Kwa-Zulu Natal has not recorded a single human death caused by rabies in a year. However, this is a small victory in the face of the continent-wide challenge. Although dog rabies is rapidly decreasing in Kwa-Zulu Natal, it is still present, and until eliminated from dogs, the likelihood of future human cases remains a stark reality.

I suggest that the road to rabies control in Africa requires a pan-African approach toward establishing sound surveillance and reporting structures that would enable proper demonstration of the expanding effect of this disease in animal and human populations in Africa. The success of such a venture is certain to be conditional to the synchronized cooperative support of OIE, WHO, and other global partners. In this regard, it is encouraging that during a high-level technical meeting in Mexico at the end of 2011 (*26*), the Food and Agricultural Organization of the United Nations together with OIE and WHO have affirmed their commitment to alignment and honing of their respective coordination mechanisms to defend against emerging diseases at the animal–human–ecosystems interfaces.
